# A workshop to enrich physiological understanding through hands-on learning about mitochondria-endoplasmic reticulum contact sites

**DOI:** 10.1152/advan.00271.2023

**Published:** 2024-09-05

**Authors:** Zer Vue, Chia Vang, Celestine N. Wanjalla, Andrea G. Marshall, Kit Neikirk, Dominique Stephens, Sulema Perales, Edgar Garza-Lopez, Heather K. Beasley, Annet Kirabo, Yelena Janumyan Doe, Desmond Campbell, Letimicia Fears, Ahmad Alghanem, Estevão Scudese, Beverly Owens, Derrick J. Morton, Clintoria R. Williams, Zachary Conley, Hinton Antentor

**Affiliations:** ^1^Department of Molecular Physiology and Biophysics, Vanderbilt University, Nashville, Tennessee, United States; ^2^Department of Medicine, Vanderbilt University Medical Center, Nashville, Tennessee, United States; ^3^Department of Internal Medicine, University of Iowa, Iowa City, Iowa, United States; ^4^Collaborative for STEM Education and Outreach, Department of Teaching and Learning, Vanderbilt University, Nashville, Tennessee, United States; ^5^King Abdullah International Medical Research Center (KAIMRC), Ali Al Arini, Ar Rimayah, Riyadh, Saudi Arabia; ^6^Laboratory of Biosciences of Human Motricity (LABIMH) of the Federal University of State of Rio de Janeiro (UNIRIO), Rio de Janeiro, Brazil; ^7^Sport Sciences and Exercise Laboratory (LaCEE), Catholic University of Petrópolis (UCP), Petrópolis, Brazil; ^8^Department of Chemistry, Cleveland Early College High School, Shelby, North Carolina, United States; ^9^Department of Biomedical Science, Kaiser Permanente Bernard J. Tyson School of Medicine, Pasadena, California, United States; ^10^Department of Biological Sciences, University of Southern California, Los Angeles, California, United States; ^11^Department of Neuroscience, Cell Biology and Physiology, Wright State University, Dayton, Ohio, United States

**Keywords:** blended learning, MERCs, mitochondria, STEMM education workshops, underrepresented

## Abstract

Physiology is an important field for students to gain a better understanding of biological mechanisms. Yet, many students often find it difficult to learn from lectures, resulting in poor retention. Here, we utilize a learning workshop model to teach students at different levels ranging from middle school to undergraduate. We specifically designed a workshop to teach students about mitochondria**-**endoplasmic reticulum contact (MERC) sites. The workshop was implemented for middle school students in a laboratory setting that incorporated a pretest to gauge prior knowledge, instructional time, hands-on activities, interactive learning from experts, and a posttest. We observed that the students remained engaged during the session of interactive methods, teamed with their peers to complete tasks, and delighted in the experience. Implications for the design of future physiological workshops are further offered.

**NEW & NOTEWORTHY** This manuscript offers a design for a workshop that utilizes blended learning to engage middle school, high school, and undergraduate students while teaching them about mitochondria-endoplasmic reticulum contact sites.

## INTRODUCTION

Physiology is a pertinent topic during students’ science, technology, engineering, mathematics, and medicine (STEMM) careers, and physiology often includes rigorous and demanding learning. Due to the difficulty being reported by students, there are ways to mitigate learning through tactile techniques that utilize novel alternative methods of teaching (1). For topics in anatomy and physiology, methods such as “Active and Engaging Learning Strategy” have increasingly been promoted in their ability to allow students to have an active role in their learning by applying nontraditional methods of learning such as poems and songs (2). In learning physiology, students often report difficulty with terminology and thorough comprehension of the concepts for their work (3), highlighting the need to improve students’ perception of physiology. Specifically, undergraduate-level students have reported greater engagement with classroom models that center on deemphasizing lecturing in favor of active and involved activities (4). Michael and McFarland emphasize in their seminal work on the 15 core concepts of physiology the importance of both comprehending the biological world and adopting unique perspectives as overarching principles of undergraduate physiology (5). Specifically, one key physiological topic is the concept of connectedness or interdependence (5), as it may be easy for students to think of organelles as independent entities as opposed to machinery involved in closely interlinked processes (3).

**Figure 1.**
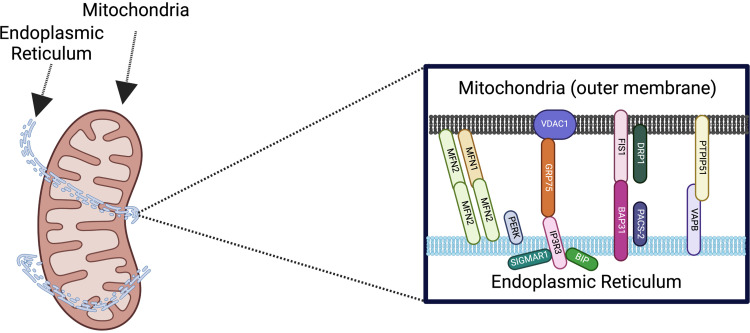
General structure of mitochondria-endoplasmic reticulum contacts and associated proteins that are discussed during the workshop. Image adapted from previous publication (7). Made with BioRender.com.

A key example of one such symbiotic relationship is known as mitochondria-endoplasmic reticulum contact sites (MERCs) (Fig. 1) (6). Mitochondria are known for their pluralistic roles including in energy production (8), while the endoplasmic reticulum has numerous roles including the synthesis and folding of proteins (9). These organelles can form close associations, which are typically less than 50 nm, that facilitate the exchange of ions and molecules (6). MERCs are now known to affect insulin stimulation, metabolism, and calcium transfer in homeostatic and disease states (9). In cases of endoplasmic reticulum (ER) stress, which is emblematic of disease states such as Parkinson’s disease, mitochondrial quality control mechanisms may be modulated by MERCs (9, 10). As a result, ER stress, which accumulates across the aging process, occurs concomitantly with mitochondrial dysfunction (11). Thus the roles of MERCs in modulating energy production across the aging process are important for future physiologists (11). However, the pleiotropic roles of MERCs are still poorly explained, with recent research demonstrating unique biochemical roles of different classifications of MERCs, such as triorganelle contacts that include peroxisomes (12).

In our experience, most classes neglect teaching about MERCs as they go outside the scope of the physiology taught. However, in the past, workshops teaching about the roles of mitochondria have been implemented across children as young as 8 to undergraduates (13, 14). We saw that MERCs represented a unique learning opportunity for students, as they could bridge several of Michael and McFarland’s core physiology concepts including Systems Integration (formerly known as interdependence), Structure ↔ Function (through teaching about different structures and functions of MERCs), and Scientific Reasoning (through a Socrative-based workshop) (5). Yet, there were no clear guidelines on how to create and teach a workshop that provides granular details on the role of MERCs. Thus we sought to create a workshop that bridges these core concepts of physiology through the incorporation of three-dimensional (3-D) modeling methods and hands-on teaching activities.

## FRAMEWORK

Here, we use blended learning in a collaborative learning environment, a technique that has been shown to improve human anatomy education in the past (15). We aim to outline an engaging workshop that teaches learners throughout different stages (middle school to undergraduate). To this end, we evaluated this workshop in a middle-school class. Specifically, we focused on middle schoolers as poor childhood recruitment in physiology fields, and broader STEMM fields, can lead to a leaky pipeline, especially for underrepresented individuals (16). Interventions occurring in seventh grade, which aim to increase students’ school participation and self-identification with school have the potential to continue to influence students as they go into eighth grade and, potentially, beyond (17). As previously and extensively discussed (18), active learning strategies are especially relevant for middle school STEMM topics yet are often neglected. In particular, middle school students’ science self-efficacy helps determine science achievement and guides students toward their college majors and careers (19, 20). However, while the workshop and evaluation were specifically targeted to middle-schoolers, the framework may be adapted to be relevant for a range of academic stages from middle school to undergraduates.

## WORKSHOP OUTLINE

**Figure 2.**
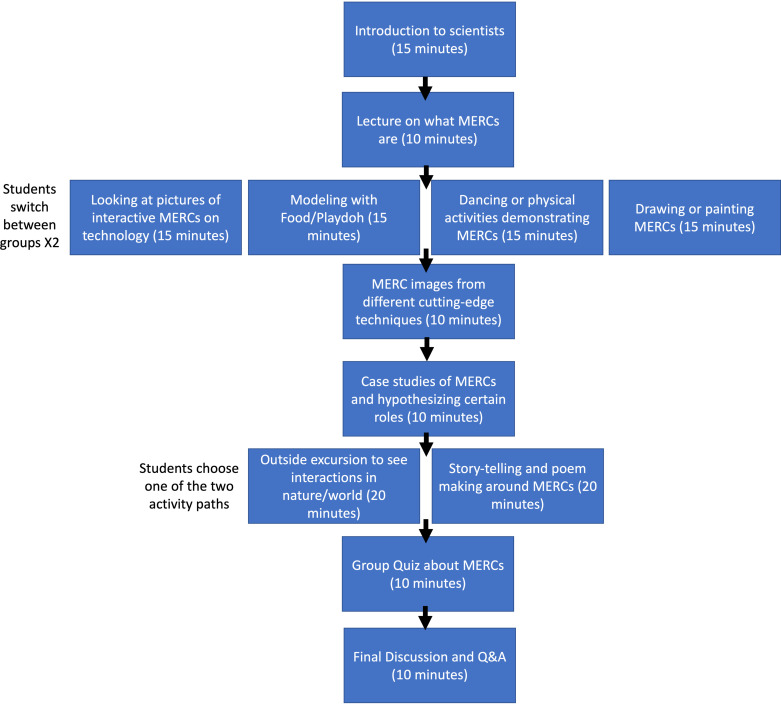
Layout of workshop. MERCs, mitochondria-endoplasmic reticulum contacts. Q&A, question and answer.

The 2-h and 45-min workshop consisted of an introduction, a lecture, experiential activities, case studies, a quiz, a discussion, and time for questions and answers (Fig. 2). This workshop was held by experts in mitochondria from across a variety of career stages (e.g., graduate students, postdoctoral fellows, assistant professors, associate professors, and professors) from a local university.

### Introduction (15 Minutes)

The 15-min introduction included an icebreaker activity where students were paired up by having either a mitochondrion taped or an ER taped on them, in an effort to form pairs of two with different functions. This can be a fun way for students to reinforce the idea behind these contact sites while engaging in collaborative activities. Once students were assigned an organelle, they passed different colored “energy balls” (representing metabolites) between designated participants (representing mitochondria and ER), symbolizing the dynamic exchange occurring in MERCs while facilitators began to introduce what MERCs are. To begin connecting the relevance of MERCs in disease, facilitators focused on certain groups to “pretend” they are in disease states. For example, they needed to pass green balls (representing calcium) if they were “MERCs in cancer.” Through interactive dialogue and decision-making, participants simulated the communication and interactions between these components. This experiential activity was supplemental to the 10-min lecture on defining MERCs.

### Lecture (10 Minutes)

The lecture included a basic PowerPoint overview of the functions and structure of MERCs. The lecture also included a brief summary of the roles of MERC, their relevance in disease states, their relevance in aging, and how they are currently studied in the current literature (6, 9, 10, 21).

### Visual Demonstrations (30 Minutes)

**Figure 3.**
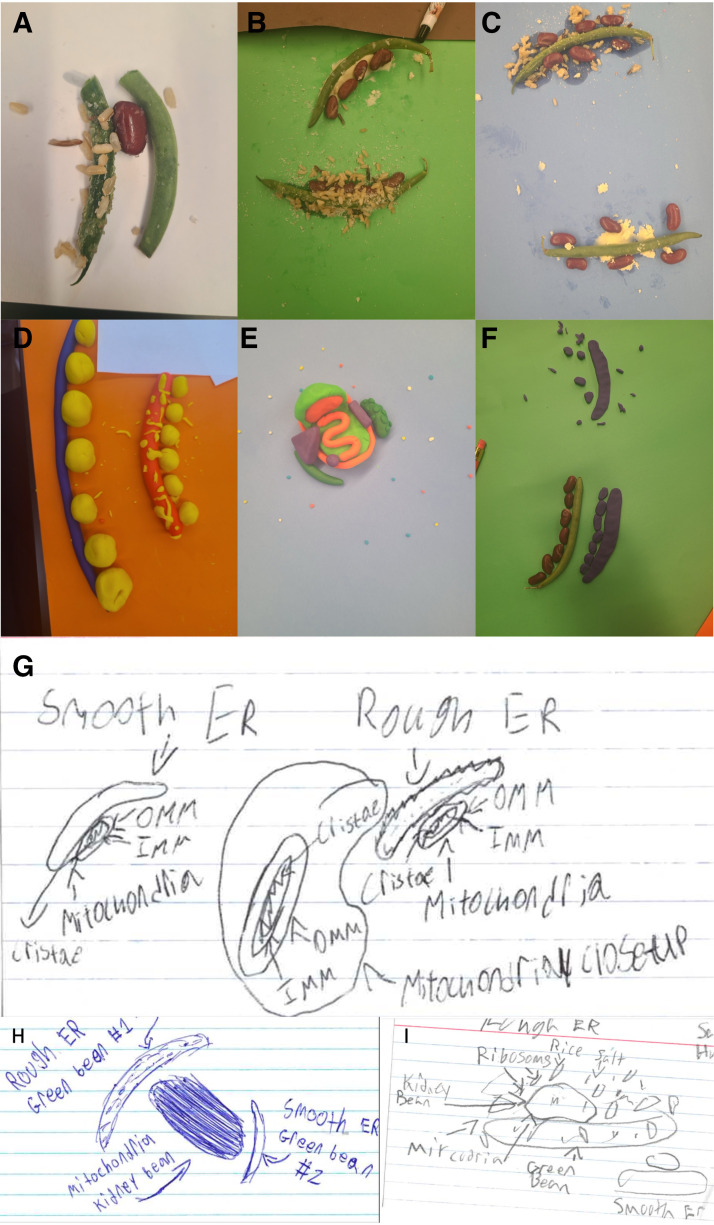
Learning with your food. *A*–*C*: string beans representing endoplasmic reticulum, rice/salt representing ribosomes, and kidney beans representing mitochondria to reconstruct mitochondria with food. *D*–*F*: students given Play-Doh to freely reconstruct different forms of mitochondria-endoplasmic reticulum contacts (MERCs). *G*–*I*: student examples of drawing mitochondria with either completely labeling (*G*) or labeling based on associations with the food items (*H* and *I*). ER, endoplasmic reticulum; IMM, inner mitochondrial membrane; OMM, outer mitochondrial membrane.

After the introduction, icebreaker, and lecture, the participants began to switch between their groups to complete experiential activities. The experiential activities (each led by 1–2 facilitators) included the following:

1)  Looking at pictures of interactive MERCs on technology. In the past, 3-D VR models installed on iPads have aided the learning of anatomy and physiology (22). In a similar vein, this interactive demonstration provided a unique and engaging perspective by showing 3-D structures of MERCs and enhancing spatial understanding.
2)  Modeling with food/playdough. The structures of MERCs were reinforced through the use of clay and 3-D models that students can utilize. This practical activity offers a tactile representation of MERCs and may help students understand their place in the cell. This activity included learning with food to represent MERCs (Fig. 3, *A*–*F*). Hands-on learning techniques have been shown to aid learning of anatomy and physiology classes in the past (23), with clay specifically helping in learning about anatomy (24, 25).
3)  Dancing or physical activity demonstrating MERCs. Pairs of participants worked together to construct a physical model representing a cellular network, incorporating mitochondria and ER components. This brief activity reminded participants of the general eukaryotic cell structure, promoted teamwork, and introduced the interconnectedness of cellular structures.
4)  Drawing or painting MERCs. During this time, students could also choose to draw or paint MERCs based on 3-D reconstructions of the structure of MERCs (example images for students were obtained from Ref. 26) (Fig. 3*G*).

These 3-D or 2-D methods of art for MERCs encouraged students to make use of both touch and sight, which improves anatomical learning (27), while also highlighting that scientific art exists and can be an option for students.

### Real-Life Applications (20 Minutes)

During this time, the workshop showcased current cutting-edge imaging techniques for MERCs, including superresolution microscopy and live-cell imaging (12, 28). Participants then performed a challenge to identify mitochondria-ER contacts and non-ER contacts across different microscopy techniques, such as transmission electron microscopy (29), 3-D electron microscopy (30), and fluorescent probes (31). Past results have shown that microscopy images can serve important roles in engaging a general audience in biological topics (32). Thus the aim of this phase was to show the transient evolution within the field and the use of technology. The use of many technologies allows for expanded opportunities for students to collaboratively guess what MERCs look like across a range of imaging techniques.

From there, facilitators presented case studies that highlighted the importance of MERCs in specific pathology processes, asking students to theorize roles such as calcium signaling, lipid metabolism, and cellular stress responses. These case studies should be flexible depending on the context and current student’s learning. Here, facilitators described a patient with heart failure, and elucidated how mitochondria are known to change in this condition, then asked participants how they think MERCs may be altered (33) and ponder how MERCs may be altered on the basis of microscopy fixation techniques (34). Participants engaged in group discussions to analyze and propose solutions to related pathologies and theorize how MERCs may be implicated in these roles.

### Learning Reinforcement (20 Minutes)

Here, facilitators offered students the chance to choose their multimodal aspect of learning:

1)  One group was dedicated to thinking about “symbiotic” associations students see in their day-to-day lives by taking a brief outside excursion. Drawing parallels between these natural phenomena and MERCs can aid students in seeing the relationship between these subcellular components and the world around them.
2)  One group of students had the chance to initiate a collaborative storytelling activity where partners contributed to building a fictional narrative involving MERCs. This can take a range of styles, from nonfiction theorization to even poems, allowing each participant to add a section to the story that incorporates scientific knowledge and creative thinking based on what has been learned thus far. During this student-led time, facilitators offered small suggestions and probing questions to encourage critical thinking and imagination.

### Quiz (10 Minutes)

From here, a multimodal interactive quiz (Table 1) using gamification techniques was administered to reinforce key concepts and test participants’ knowledge. Gamified techniques, including nominal rewards and points for correct answers, encouraged motivation, engagement, and performance while allowing for the groups of students to work together and critically discuss topics brought up (35). Here, we performed the quiz on paper to record student answers for postworkshop analysis. However, this quiz may be performed through any preferred technique, including Kahoot or other virtual platforms.

**Table 1.** Questions asked to students (fill in the blank)

Questions Asked
What is a MERC? What is Rough ER? What is Smooth ER? What is TEM? What is 3DEM? Name a MERC protein. What is the distance between mitochondria and ER? What goes between mitochondria and ER? What happens when a MERC is not active? What happens when a MERC interacts with other organelles?

3-DEM, 3-dimensional electron microscopy; ER, endoplasmic reticulum; MERCs, mitochondria-endoplasmic reticulum contacts; TEM, transmission electron microscopy.

### Conclusion (10 Minutes)

To conclude the workshop, the facilitators, who in our case were experts in the field of mitochondria-ER contact sites, briefly shared their experiences, insights, and personal journeys in the STEMM field. Participants were able to ask questions, engage in discussions, clarify any points of confusion, and network with individuals in the field if they desired future summer opportunities.

## METHODS

### Institutional Review Board Approval

The study was approved by the Institutional Review Board (IRB) of Vanderbilt University and the IRB for the Day of Discovery Program (No. 160120). The workshop and associated data collection were approved by the Institutional Review Board of Vanderbilt University.

### Workshop

This workshop was performed in accordance with the framework above. Workshop data were gathered from three middle school classes participating in a unique “pull-out” program in which they leave their local public school and attend classes and workshops at Vanderbilt University. In total, this workshop was run three times, with each class having ∼28 students for a total of 85 students. Two scientists from a local academic institution delivered the workshop, and three additional scientists assisted students in the project, for an approximate one-to-five instructor-to-student ratio. The moderators collected the students’ responses and anonymous written qualitative feedback following the workshop, while an anonymous pretest was administered before the onset of the workshop.

### Participants

All students were free to choose whether or not to participate in the quiz or have their answers utilized for analysis. Thus, despite 85 total participants, only 46 are included in analysis of pre- and posttest responses. However, of all 85 students, 49% were male, 46% were female, and the remaining 5% either were nonbinary or did not answer. Students also were self-reported as 59% White, 20% Black, 13% Hispanic, 3% Asian and/or Pacific Islander, 2% Middle Eastern, and 2% “no answer.”

## RESULTS

Qualitative feedback was received from 16 of the 85 total middle school students who participated in the study. Feedback was generally positive (Table 2). Specifically, multiple students said they loved the activity and found it fun and informative. The most common positive adjective to describe this workshop was fun, with individuals especially enjoying the hands-on activities including modeling and drawing MERCs, especially when it came to using food. Moreover, nine participants expressed their keen support of experiential activities as an alternative method of learning. Although the experiential activities were deemed as most helpful, there were also some reported issues. Five participants greatly enjoyed the activities but wished they had more time for them. Other students reported issues of minor inconveniences with the materials, such as disliking Play-Doh or the specific food items used. However, the most common running theme alluded to time management. Participants reported there was too much information being delivered in a short time with individuals feeling confused as a result.

**Table 2.** Written quotes of student feedback

Positive	Negative
I liked the experiment we did, that was fun. I thought that this lesson was very interesting & informing. I likes the interactiveness of the diagram I did learn a lot of new things. I really liked how the information was relevant and very new. I liked modeling the ER. I liked the food. Fun. Food was good (see back). It was fun. Cool, fun, and engaging. The modeling of the ER was interesting, as well as the explanation. Today’s lesson was good, and it was explained very well. I thought the interactive, hands-on part was fun and it was interesting to hear about reverse aging stuff. It was a[n] 8 out of 10 lesson, overall it was great and fun. I learned much more than in normal school: answered our questions and explained it well. The lesson was good. I liked how we had a hands on project – it explained it well.	I wished we would have more time for clay modeling. Something that could be improved would be to make it less confusing because I got a little lost a few times. It did go a little fast & I was a bit confused. The lesson was interesting but kind of hard to understand. I also think that it was expected to have a lot of prior knowledge, but nobody really did. I think you could move a little slower when explaining things. I did not like the test. None. Basically, all good. Would have been nice to have been told what they [MERCs] represented. The clay stuff made my hands sticky. The tests were boring, but nothing to help that, I suppose. N/A N/A I honestly thought I personally need more information [because] I wasn't feeling most confident with the quiz after. Went a little fast. I just got confused because the oil is what makes something soft/smooth. One thing that was confusing was that there was a ton of information, all in a row, so it was hard to understand and process.

Students were asked to name one positive (if any) and one negative (if any) aspect of the workshop; *n* = 16. MERCs, mitochondria-endoplasmic reticulum contacts; N/A, not applicable.

**Figure 4.**
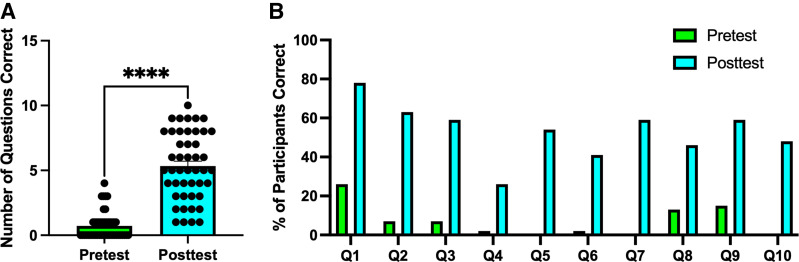
Student results in between pre- and posttests. *A*: comparison of total correct questions (Q; out of 10) in individuals comparing pretest and posttest. *B*: individual comparison of each question (Table 1) with percentage correct before and after the workshop. ****P < 0.0001, paired *t* test, as determined via Prism to evaluate statistics; *n* = 46.

We also conducted an individual pre- and posttest with questions relating to MERCs and their functions (Table 1). Of the 46 individuals who participated in these pretests, on average, individuals got 0.71 questions (out of 10; 7.1%) correct on the pretest compared with 5.33 questions (53.3%) correct on the posttest, representing a significant improvement (Fig. 4*A*). This was marked by an improvement in the percentage correct for every single question (Fig. 4*B*). In the pretest, multiple questions were not answered correctly by a single student (e.g., *questions 5* and *10*). The question with the highest correct rate in the pretest (*question 1*) was answered correctly by only 26.1% of participants. In comparison, the question with the lowest correct rate in the posttest (*question 4*) had 26.1% of individuals answer it correctly. In the posttest, the question with the highest correct rate (also *question 1*) had 78.3% of participants answer it correctly.

## DISCUSSION

This activity aimed to provide a multimodal mechanism to teach middle-school students about MERCs and increase their interest in STEMM. Based on our results, students generally enjoyed the workshop, although future improvements such as the level of detail, which can be tailored based on the grade of the learners may be made to create a more robust workshop.

### Improvements for Future Workshops

There may have been some confusion around the questions asked in the quiz. For example, for *questions 2* and *3* (Table 1), some individuals simply spelled out the term ER, rather than explaining their purpose, which is what qualified as a correct answer. Thus these questions may be better reworded to “Describe the purpose of smooth and rough ER.” Other questions had multiple potential correct answers depending on students’ attention; for example, “What goes between mitochondria and ER?” had individuals commonly describe the materials (e.g., calcium and lipids) or MERC proteins, both of which were marked as correct.

Beyond this, in the future, the workshop should aim to either be held across a longer period or cover less information. The present workshop was 2 h and 45 min, which should be considered an absolute minimum amount of time for the information presented. If the workshop were to be shortened, we found that the “Real-Life Applications” sections, while being pertinent, had the lowest student engagement due to the less hands-on nature of it. While qualitatively many students mentioned they felt confused or overwhelmed by the content delivered, many students still understood much of the content delivered, with even questions asking about specific MERC proteins having a relatively high percentage answered correctly. Given the participant feedback, in the future, we would recommend the workshop to be 3–4 h. This recommendation could aid in the meaningful exploration of alternative learning. Yet, this longer length may cause difficulties in student attention if not supplemented with additional breaks. Alternatively, for several weeks before this workshop, students may be taught adjacent topics including calcium signaling and cellular stress response to be more prepared for the topics in the workshop.

Nonetheless, it must be noted that there is a common phenomenon in which individuals in STEMM, regardless of actual ability, may choose to drop out of the pipeline due to feelings that the topics are too difficult for them (36). This can especially be an issue for underrepresented students (37). Thus, future recommendations support the modification of questions to best align with diverse populations and to ensure students do not feel like STEMM in general is too difficult.

### Future Potential Expansion

This workshop should prioritize increasing engagement across a range of methods and can utilize resources in the environment being administered. For example, if students have a cooking class, this workshop may be expanded to include a cooking class that focuses on foods and recipes associated with energy metabolisms, such as those rich in protein, antioxidants, vitamins, and minerals that facilitate metabolism (38, 39). Cooking classes have been shown to improve the learning of medical students regarding nutrition while also promoting nutritional awareness (40, 41). This lesson can be related to MERCs by connecting these nutrients to mitochondrial function, and, therefore, potentially the structure of MERCs. Beyond this, to be even more hands-on, the dynamic fusion of mitochondria could be exemplified by the creation of desserts; for example, students can make a “mitochondrial” cake with inner fillings (to represent cristae) and differently colored outside frosting (to represent the outer membrane). By combining and cutting these cakes in half, students can imagine how mitochondria undergo dynamic cycles of fusion and fission (42). Another group of students can make intricate and interconnected patterns with gelatin or fruit to represent ER. When combined, students can see dessert transforming into a unified creation, akin to MERCs, while facilitators can discuss how mitochondrial fusion might affect MERC formation, such as in disease states and across aging. Furthermore, if a 3-D printer or modeling class is already at the institution, this can allow students to work on modeling and printing MERCs for the activity themselves. Thus this blended learning workshop should prioritize using the resources available at the institution to reinforce learning.

### Adaptations for Increased Rigor for High School and Undergraduate Workshops

This workshop is best adapted for middle schoolers and high schoolers. However, it may also be adapted for undergraduates as a more fun opportunity that may aid in reducing burnout. If delivered to high schoolers or undergraduates, the content should be scaled up appropriately; for example, the workshop can discuss more novel forms of WrappER (12). Beyond this, the technology underlying techniques such as serial block-face scanning electron microscopy (43) may be discussed to ensure even students who may not be interested in mitochondrial study can learn about cutting-edge techniques in scientific fields. Furthermore, a discussion of how mitochondrial shape may change in different disease states (33), aging in tissue (44–46), and other factors that influence mitochondrial shape, as well as a critical discussion of the interplay between mitochondrial and ER shape, can be useful in these advanced workshops. Furthermore, we have developed workshops for the undergraduate level specifically targeting underrepresented individuals and seeking to improve career and professional development (46–50). In the future, it may be worthwhile to evaluate a comprehensive framework of workshops that bridges together science-focused workshops, like this one, and workshops based on graduate school preparedness.

### Conclusions

While the primary intent of this workshop is to inform, the secondary is to cause students to learn how much fun one can have in learning about these topics. Past results have shown that utilizing art and other hands-on activities for anatomy learning is not only more enjoyable for students but also aids their learning and increases the likelihood they will engage in out-of-hours learning opportunities (51, 52). Specifically, multimodal workshops are underutilized for the teaching of mitochondria, as past studies have shown they significantly aid in student self-confidence, and if given the option to pursue a multimodal method, 97% of students do so (53). Here, we found that middle schoolers positively responded to this workshop, particularly believing it was fun and interesting, albeit slightly confusing in its current format. We believe that the workshop laid out here and with the suggestions provided based on our pilot study may cause students to not only have increased retention; additionally, they may also take a deeper interest in how physiology can be learned and the general interconnectedness of mitochondria and other organelles.

## DATA AVAILABILITY

Data will be made available upon reasonable request.

## GRANTS

Support was provided by The United Negro College Fund (UNCF)/Bristol-Myers Squibb E.E. Just Faculty Fund, Career Award at the Scientific Interface (CASI Award) from Burroughs Welcome Fund (BWF) ID No. 1021868.01, BWF Ad-hoc Award, National Institutes of Health (NIH) Small Research Pilot Subaward to 5R25HL106365-12 from the NIH PRIDE Program, NIH Grant DK-020593, and Vanderbilt Diabetes and Research Training Center for Diabetes Research and Training Center (DRTC) Alzheimer’s Disease Pilot & Feasibility Program Chan Zuckerberg Initiative (CZI) Science Diversity Leadership Grant 2022-253529 from the Chan Zuckerberg Initiative DAF, an advised fund of Silicon Valley Community Foundation (to A.H.J.).

## DISCLAIMERS

The funders had no role in the study design, data collection and analysis, decision to publish, or preparation of the manuscript.

## DISCLOSURES

No conflicts of interest, financial or otherwise, are declared by the authors.

## AUTHOR CONTRIBUTIONS

A.H. conceived and designed research; Z.V., C.V., Y.J.D., D.C., L.F., D.J.M., Z.C., and A.H. performed experiments; Z.V., C.V., C.N.W., A.G.M., K.N., D.S., S.P., E.G.-L., H.K.B., A.K., Y.J.D., D.C., L.F., A.A., E.S., B.O., D.J.M., Z.C., and A.H. drafted manuscript; Z.V., A.K., Y.J.D., D.C., L.F., A.A., E.S., B.O., D.J.M., C.R.W., Z.C., and A.H., edited and revised manuscript; C.R.W. and A.H. approved final version of manuscript.
